# A novel connexin 50 (*GJA8)* mutation in a Chinese family with a dominant congenital pulverulent nuclear cataract

**Published:** 2008-03-04

**Authors:** Ming Yan, Chenling Xiong, Shui Qing Ye, Yongmei Chen, Min Ke, Fang Zheng, Xin Zhou

**Affiliations:** 1 Department of Ophthalmology, Zhongnan Hospital, Wuhan, China; 2 Center for Gene Diagnosis, Zhongnan Hospital, Wuhan, China; 3 Department of Surgery and Department of Molecular Microbiology and Immunology, University of Missouri School of Medicine, Columbia, MO

## Abstract

**Purpose:**

To identify the genetic cause responsible for the autosomal dominant hereditary cataract in a Chinese family.

**Methods:**

A whole family of a proband who has a dominant congenital pulverulent nuclear cataract was recruited into Zhongnan Hospital. The lenses of patients were observed by a slit-lamp microscope, and the lenses of the proband’s mother were analyzed by scanning electron microscopy. Mutation screening was performed in the cataract candidate genes coding for crystallins and connexin 50 by sequencing of polymerase chain reaction (PCR) products amplified from blood leukocyte DNA samples of eight family members. The identified mutation was then investigated in other participated family members, 200 normal controls, and 40 senile cataract patients by the restriction fragment length polymorphism (RFLP) method.

**Results:**

The structure of the lens opacities of the proband’s mother is puffy, and the fibers are tangled under a scanning electron microscope. A novel C>T transition at nucleotide position 827 was determined in the connexin 50 (*GJA8)* gene. This mutation led to a serine (S) to phenylalanine (F) amino acid substitution in amino acid position 276 where the secondary structure prediction suggested a helix replaced by a sheet. And the mutation was neither found in the 200 controls nor in the 40 senile cataract patients.

**Conclusions:**

A novel *GJA8* gene mutation was found to be associated with hereditary cataract in a Chinese congenital cataract family.

## Introduction

Cataract accounts for about 41.06% of blindness and is the leading cause of visual impairment in China [1]. Furthermore, the incidence of cataract continues to rise. So far, only surgical intervention could prevent or delay the occurrence and development of cataract. New therapeutic modalities are needed, but their development has been impeded by the incomplete understanding of cataract formation mechanisms. Although there are multiple factors involved in the etiology and development of cataract, accumulating evidence indicates that genetic background plays an important role in the whole process. Research on hereditary congenital cataracts led to the identification of several classes of candidate genes [2] that encode proteins such as the crystalline proteins (CRYAA, CRYAB, CRYBA1/A3,CRYBB1, CRYBB2, CRYBB3, CRYGC, CRYGD, and CRYGS) [3,4], the gap junction protein [5], major intrinsic protein (MIP/MIP26) [6], lens integral membrane protein 2 (LIM2/MP19), the beaded filament protein (BFSP2) [7], paired-like homeodomain transcription factor 3 (PITX3) [8], heat shock protein (HSF4) [9], and galactokinase [10]. Knowledge of the structures and functions of those candidate genes as well as the pathophysiology effect of their disease-associated mutations on their functions will provide hints for understanding the mechanisms of cataracts.

In this paper, we report a novel S276F (827C>T) mutation in the connexin 50 gene in a Chinese five-generation family with the pulverulent nuclear cataract. This is a missense mutation that has not been reported previously with an inherited cataract.

## Methods

### Family data

**Figure 1:**
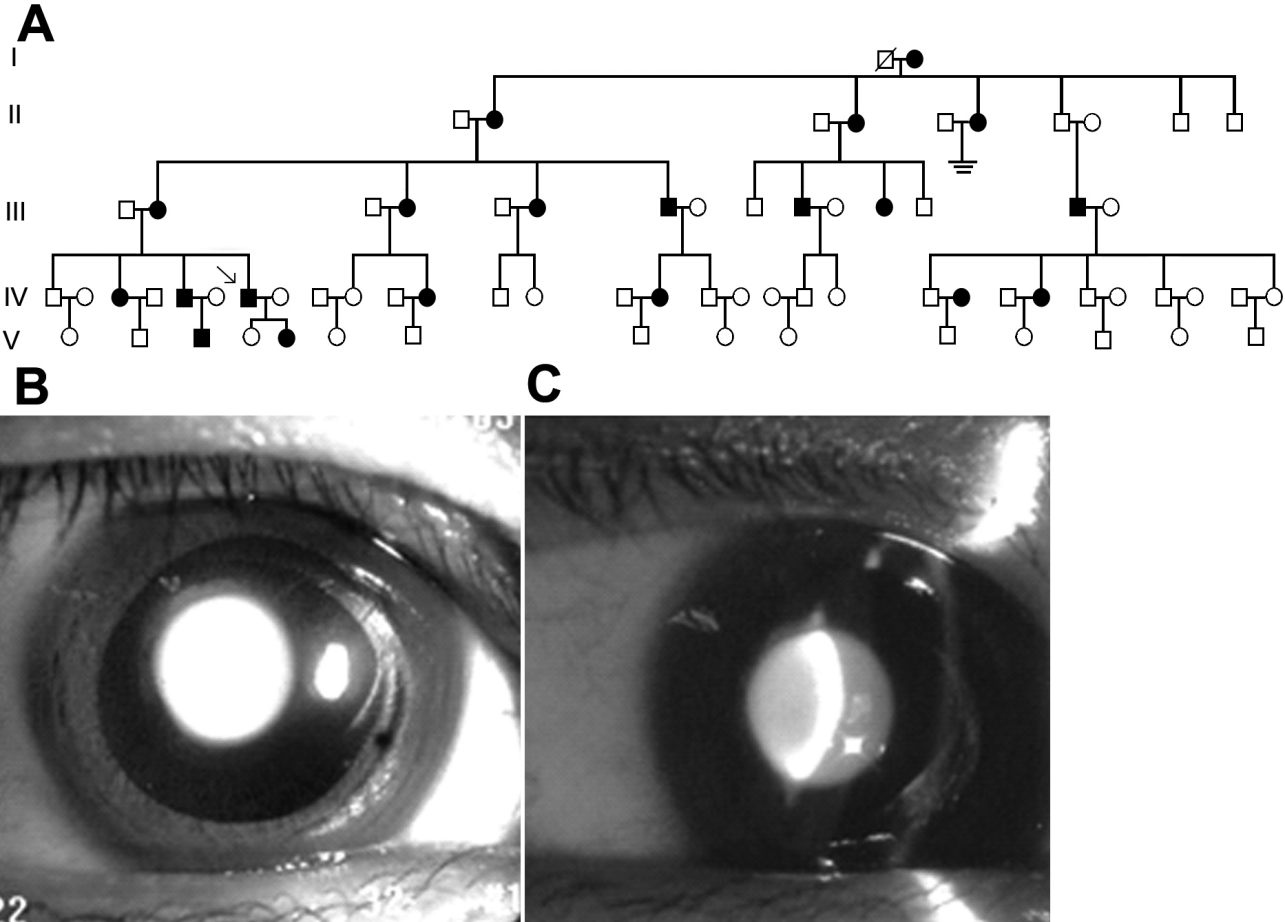
Family pedigree in this study and lens photograph of the proband (IV: 7). The pedigree is a large five-generation Chinese family (**A**). Females are represented by circles and males by squares. Ophthalmologist confirmed affected individuals are colored black. The phenotype exhibits the appearance of pulverulent nuclear cataract (**B**). The optical section shows lens opacities in the proband. The fetal nucleus and the embryonal nucleus both display white granular opacities (**C**).

A Chinese family was recruited into Zhongnan Hospital, Wuhan, China. The pedigree is presented in Figure 1A. Ethical approval for this study was obtained from the Zhongnan Hospital Research Ethics Committee. Informed written consent was obtained from all adult individuals and the parents of children under 16 years of age.

The proband is a 39-year-old man. He was diagnosed with a congenital pulverulent nuclear cataract (Figure 1B,C), and the cataract began around seven years of age with progression. Full ophthalmic assessment was performed in the participated subjects. Ten affected and sixteen unaffected members in the family participated in the study.

### Electron microscope analysis

The lenses of the proband’s mother were removed during operation and prefixed in a solution containing 2% glutaraldehyde at room temperature for two days. The lenses were washed in 0.1 M phosphate buffer saline (pH 7.2) three times, postfixed in 1% aqueous OsO_4_ for 2 h, and then washed again three times in 0.1 M phosphate buffer saline (pH 7.2). The samples were replaced in isoamyl acetate for 20 min before being dehydrated in a series of ethanol-water washes (25%, 50%, 75%, two 95%, and three 100% ethanol). Finally, samples were dried using a critical point drying apparatus, firmly mounted, and sputter coated with a thin layer of gold before examination under a HITACHI S-570 electron microscope.

### DNA isolation

The genomic DNA was extracted from venous blood leukocytes of subjects using the improved NaI method [11].

### Mutation detection

Mutation screening in the known candidate genes for hereditary cataracts such as *CRYAB, CRYAA, CRYBB2, CRYGC, CRYGD,* and *GJA8* was performed by polymerase chain reaction (PCR) amplification of the exonic regions of those genes and by direct DNA sequencing of those PCR amplicons. The sets of primer pairs used in the PCR amplifications are listed in Table 1.

**Table 1:** Primers used to screen mutations.

**Gene symbol and accession number**	**Sequence**	**Amplified fragment**	Annealing temperature*	**Size**
***GJA8*** GI:6942063	F:CGGGGCCTTCTTTGTTCTCTAGTCC R:AGGCCCAGGTGGCTCAACTCC	The first part of exonic fragment	60	877 bp
	F:CAGCCGGTGGCCCTGCC R:GTTGCCTGGAGTGCACTGCCC	The second part of exonic fragment	69	907 bp
***CRYGC*** GI: 37551287	F:TCAATCATATAGACAGAGCCA R:ATGTCCATCTAACCCTTAGGT	Exons 1 and 2	55	784 bp
	F:AATGACAATTCCATGCCACA R:CCCACCCCATTCACTTCTTA	Exon 3	55	534 bp
***CRYGD*** GI: 37551287	F:TGATAGCAATCCGAATACTCCA R:GGGTAATACTTTGCTTATGTGGGGAG	Exons 1 and 2	55	776 bp
	F:GTCCTCACCAAGCTGGACTG R:CCATTTGCCTCGTGTGTGTA	Exon 3	55	496 bp
***CRYBB2*** GI: 16168698	F:CACTGTGTCCAAGGTCACACAGCTAAGC R:CCCCTCGTTCACCCTCCCATCA	Exon 6	69	506 bp
***CRYAA*** GI: 37559293	F:CTCCAGGTCCCCGTGGTA R:AGGAGAGGCCAGCACCAC	Exon 1	60	251 bp
	F:CTGTCTCTGCCAACCCCAG R:CTGTCCCACCTCTCAGTGCC	Exon 2	65	220 bp
	F:GGCAGCTTCTCTGGCATG R:GAGCCAGCCGAGGCAATG	Exon 3	60	309 bp
***CRYAB*** GI: 27540935	F:AGGATGAATTACCCGGACAGAAAG R:ACCCCTGATCCCGACTGTTAT	Exon 2	65	360 bp
	F:TGAGTTCTGGGCAGGTGATAATAGTT R:AGCTTGATAATTTGGGCCTGCC	Exon 3	60	391 bp

F: forward primer, R: reverse primer. The genomic DNA was amplified in a 25 μl reaction volume with 10 pmol forward primers and reverse primers. The asterisk (*) indicates that the PCR conditions were the following: denaturation at 95 °C for 5 min followed by 35 cycles of denaturation at 95 °C for 45 s, annealing at the Annealing Temp for 30 s, and extension at 72 °C for 30 s with the last extension for 10 min at 72 °C.

To amplify the second fragment of the exonic region in *GJA8*, genomic DNA extracted from the proband was amplified in a 25 μl reaction volume. The PCR conditions are as follows: denaturation at 95 °C for 5 min followed by 35 cycles of denaturation at 95 °C for 45 s, annealing at 69 °C for 30 s, and extension at 72 °C for 30 s with the last extension for 10 min at 72 °C. The PCR products of 907 bp were purified and sequenced using an ABI Genetic Analyzer 3730 (Invitrogene Ltd, Shanghai, China). The sequencing results were analyzed using Chromas 2.3 and blasted in the NCBI database.

### Restriction analysis

Segregation of the mutation in the other participating family members was determined by the polymerase chain reaction-restriction fragment length polymorphism (PCR-RFLP) method with the restriction enzyme Hpy188I (New England Biolab, Beijing, China). We redesigned a pair of primers using Primer Premier 5.0 to amplify the DNA fragment of 239 bp that contained the mutation site based on the published human connexin 50 DNA sequence (GenBank accession number AF217524, GI: 6942063). The primer sequences were as follows: forward primer (GJA8ZF1), 5′-TCA TCC TGT TCA TGT TGT CTG TGG C −3′; reverse primer (GJA8ZF2), 5′–AAC CTC GGT CAA GGG GAA ATA GT**C** G-3′. The C base (underlined) at base pair 24 of the reverse primer was mutated from G to C to produce a recognition site for the restriction enzyme Hpy188I (TCNˇGA). If there was a mutation of C to T, the recognition site of restriction enzyme Hpy188I would have been abolished. PCR conditions were the following: 1 cycle at 95 °C for 5 min; 35 cycles at 95 °C for 45 s, 60 °C for 45 s, and 72 °C for 45 s; then 1 cycle at 72 °C for 10 min. The PCR product was digested with 10 U of Hpy188I at 37 °C for 12 h.

The digested PCR products were separated on 8% polyacrylamide gel (acrylamide: bisacrylamide=29:1) in 1×TBE for 1.5–2 h at a constant voltage of 100 V. The PCR products that were amplified from samples of individuals with the wild-type genotype were digested into fragments of 215 and 24 bp while the presence of the 827C>T mutation resulted in fragments of 239, 215, and 24 bp.

**Figure 2:**
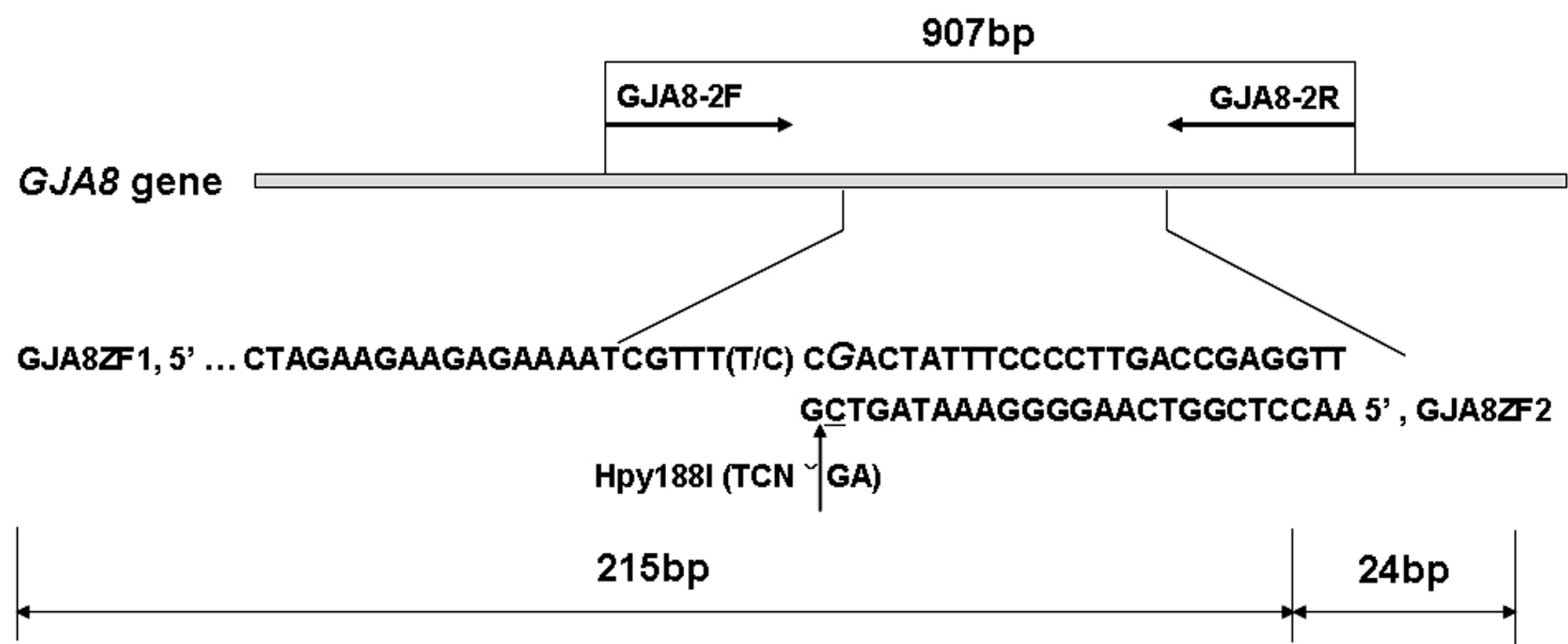
Schema for the mutation screening in *GJA8*. One pair of primers (GJA8–2F and GJA8–2R) were used to amplify the big fragment of the exon in *GJA8*, and the other pair (GJA8ZF1 and GJA8ZF2) was used to detect the mutation by RFLP.

The schema for the mutation screening in *GJA8* by PCR-sequencing and RFLP methods is shown in Figure 2.

### The secondary structure prediction

The secondary structure of mutant and wild-type amino acid sequences were analyzed by Antheprot 2000 V 6.0 software.

## Results

### Electron microscope analysis

**Figure 3:**
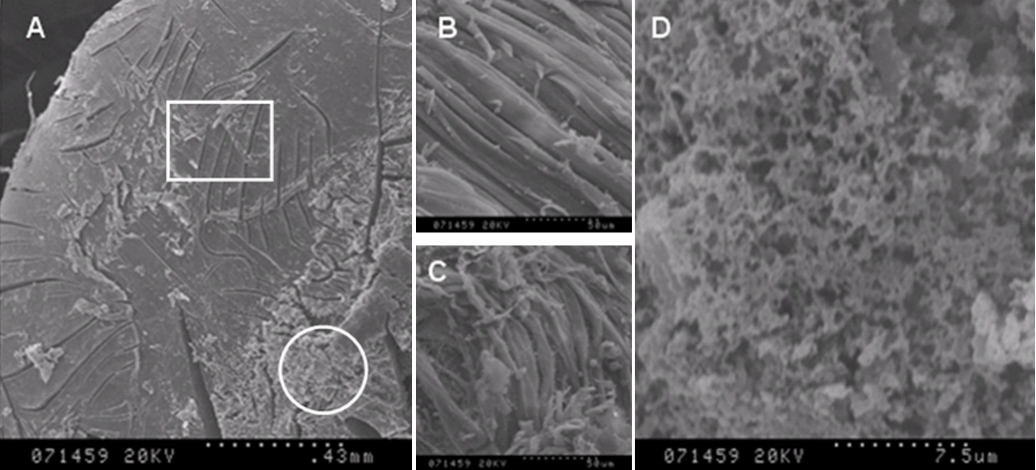
Cross section of the lens of the proband’s mother (III: 2) under the scanning electron microscope. The normal lens tissue was shown in a square while the opacity was shown in a circle (**A**). The parallel aligned strap fiber cells are represented in the longitudinal section (**B**). The structure of the opacities are puffy and irregularly aligned while floccules were observed and the fiber is tangled (**C,D**).

In the proband’s mother, there are opacities in the fetal nucleus and embryonal nucleus. The structure of the opacities is puffy, and the fibers are tangled (Figure 3).

### Mutation screening

**Figure 4:**
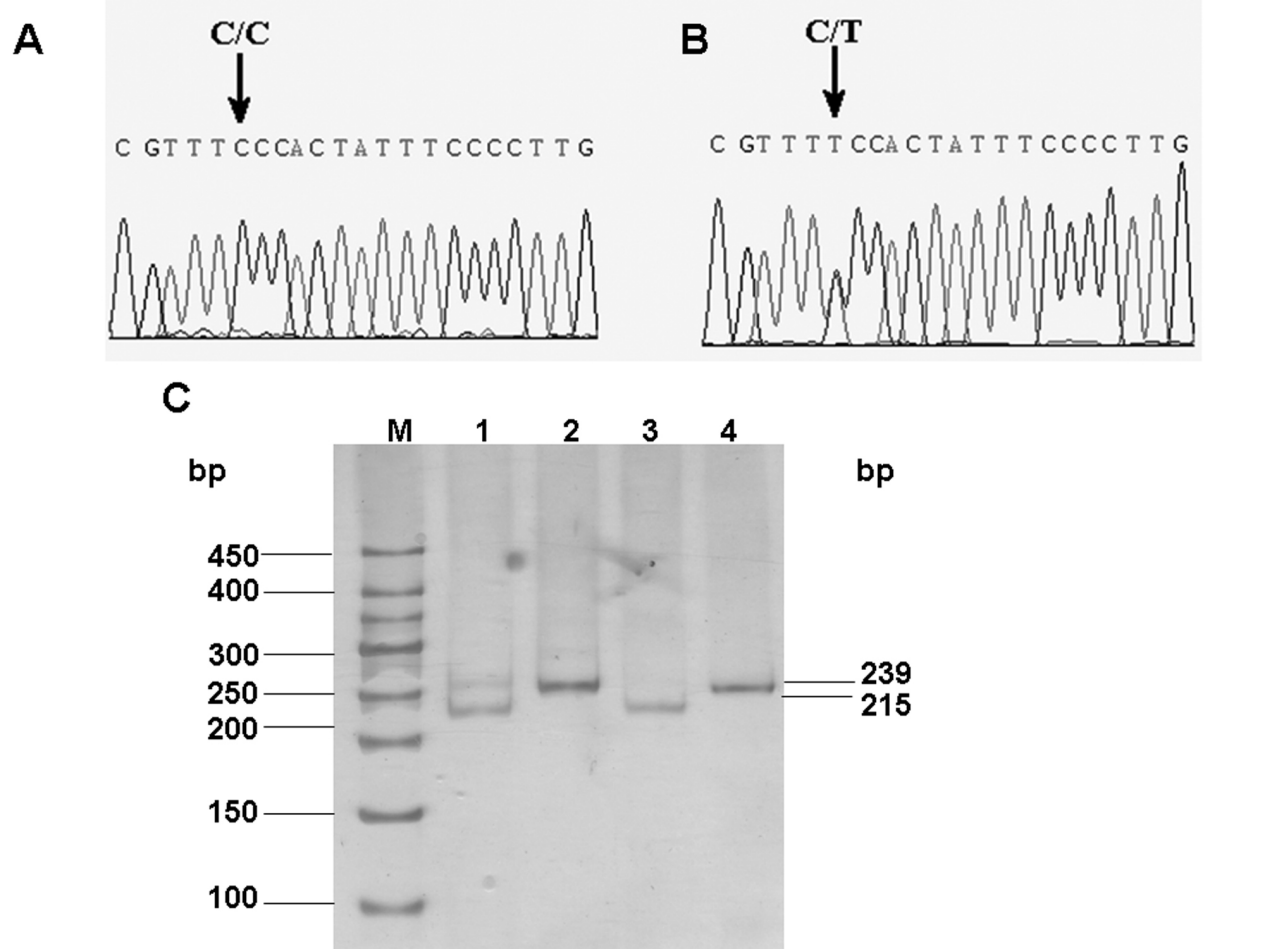
Mutation analysis of *GJA8*. Sequence chromatograms of the wild-type allele (**A**) demonstrate a nucleotide sequence encoding a serine (Ser) at codon 276, and the chromatograms of the mutant allele (**B**) demonstrate a C to T transition resulting in the substitution of serine (Ser) by phenylalanine (Phe). Confirmation of segregation of the S276F mutation was given by the PCR-RFLP method (**C**). M is the DNA ladder; Lane 1 shows the digestions of the PCR products amplified from samples of the patients by Hpy188I. Lane 2 and 4 show the PCR products amplified from samples of one patient or the unaffected member in the pedigree. Lane 3 illustrates the digestions of the PCR products amplified from samples of the unaffected family member by Hpy188I. The mutant primer results in the gain of *Hpy*188I sites producing digested fragments of 215 and 24 bp with wild-type *GJA8* alleles, and the mutation in the *GJA8* gene leads to an abolition of this site, remaining undigested (239 bp).

**Figure 5:**
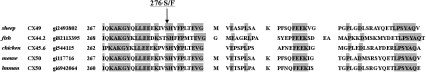
A multiple alignment of amino acid sequence of GJA8. A multiple alignment of amino acid sequence of GJA8 with different connexin species. Identical residues are highlighted with a dark background. The amino acid marked with an arrow is the mutant amino acid at codon 276, where it is highly conserved throughout all connexins.

Sequencing of the exonic region of *GJA8* in eight participants (three affected and five unaffected) showed a heterozygous mutation 827C>T in all the affected members (Figure 4B). This mutation was also screened in other participated members in the five-generation Chinese family by restriction enzyme digestion with *Hpy*188I (Figure 4C). The mutation was segregated with the phenotype of all affected family members who participated in this study. It was not present in the unaffected family members or in the 200 controls. Furthermore, 40 Chinese senile patients with age-related nuclear cataract were screened, and the mutation failed to be detected. These results suggested that this 827C>T mutation was associated with the dominant congenital pulverulent nuclear cataract. This nucleotide transversion replaced an evolutionarily conserved serine (S) with phenylalanine (F) at amino acid position 276 in the cytoplasmic COOH-terminal domain of connexin 50 (Figure 5).

### The secondary structure prediction

**Figure 6:**
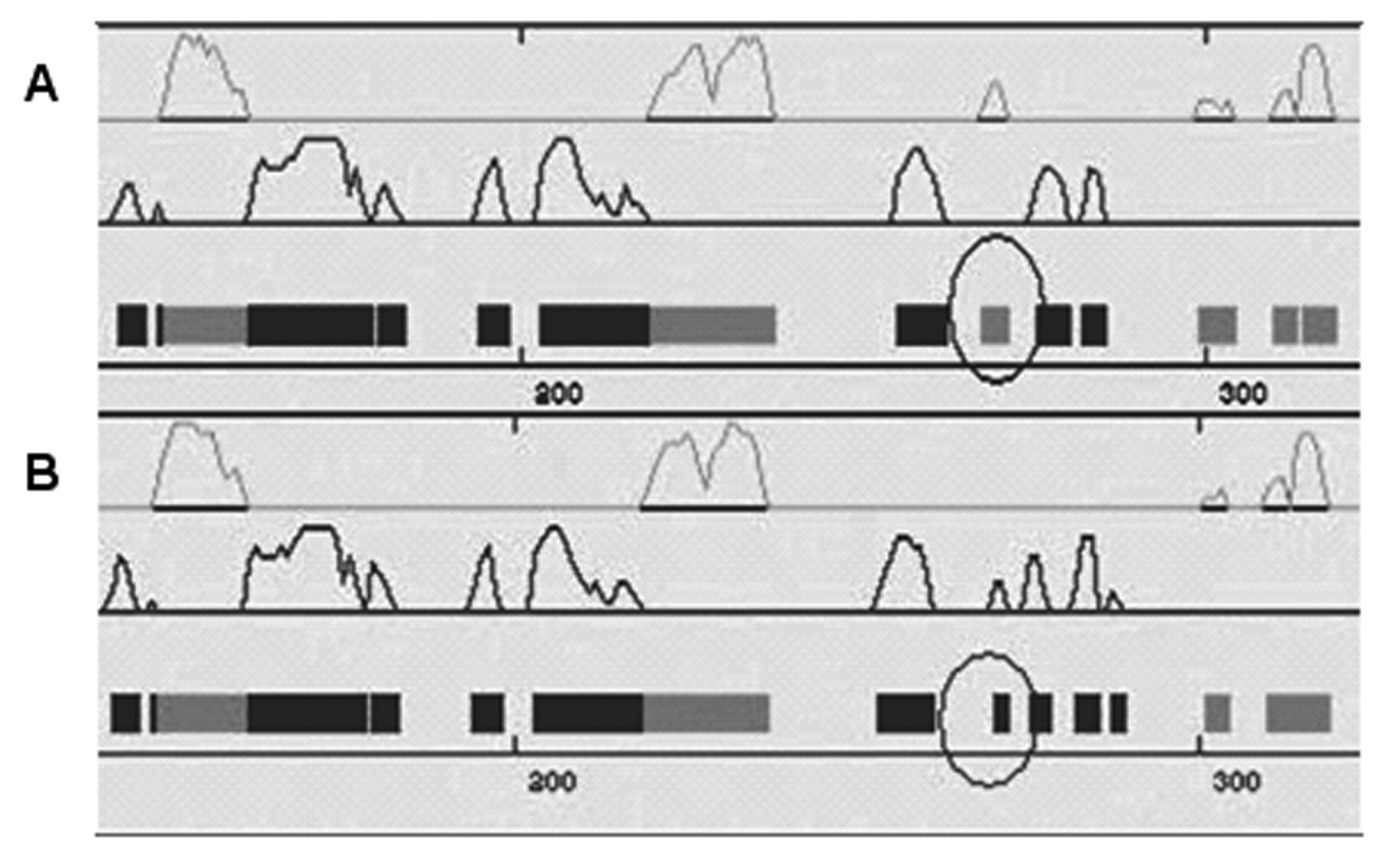
The predicted secondary structures of the mutant and the wild type amino acid sequences. The predicted secondary structures of the mutant amino acid sequence (**A**) and the wild-type amino acid sequence (**B**) is shown. The target sequences were labeled by a black circle, which indicated that there is a helix in the wild-type replaced by a sheet in the mutant type. Grey: helix; Black: sheet.

The mutation S276F leads to the replacement of an original helix by a sheet, a significant difference in coding position 276 of the secondary structures of gap junction protein 50 (Figure 6).

## Discussion

Cataracts are defined as lens opacities. The lens is composed of post-mitotic fiber cells. The final differentiation of fiber cells includes the complete removal of all cellular organelles and its inability to undergo protein synthesis, DNA replication, and RNA transcription [12]. All lens fiber cells are retained in the aqueous humor; they are completely avascular in lenses. The fiber cells are connected by intercellular gap junctions. Gap junctions are composed of hundreds of gap junction channels and provide pathways for the exchanges of small molecules such as amino acids, calcium, cyclic adenosine monophosphate (cAMP), inositol triphosphane (IP3), etc. in different vertebrate tissues such as the lens, brain, heart, and liver [13-15]. Gap junction channels in the lens maintain the homeostasis required for lens transparency. A gap junction channel is oligomerized by 12 connexin proteins. There are various gap junction proteins, and among them, connexin 46 and connexin 50 (Cx50 or GJA8) are major lens connexins [15]. Cx50 contains four transmembrane domains, three intracellular regions (the NH_2_-terminus, a cytoplasmic loop, and the COOH-terminus), and two extracellular loops (E1 and E2) [16]. The coding region of *Cx50* is completely contained within one exon. The size of the *Cx50* translation region from the transcript start codon to the terminating codon TGA is 1,299 nucleotides. It encodes a polypeptide containing 432 amino acids with a molecular weight of 48,171 daltons [17]. The connexin 46 genes and *Cx50* genes, when disrupted in the mouse, lead to congenital cataract [18,19]. Mutations in connexin 46 (α3, *Gja3*) and connexin 50 (α8, *Gja8*) genes lead to congenital cataract in human and mice [20,21]. Xia et al. [22] provided evidence that different mechanisms modulated by various gap junction proteins influenced the formation of primary and secondary fiber cells during lens development. It was concluded that mutants in diverse connexins caused different phenotypes of cataract.

The nuclear pulverulent cataract described in our study was associated with a mutation in *GJA8*. The 827C>T transversion resulted in a serine (S) to phenylalanine (F) amino acid substitution in the cytoplasmic COOH-terminal domain of connexin 50. This non-conservative mutation replaces α-amino-β-hydroxy-propionic acid with α-amino-β-phenyl-propionic acid; the hydroxy was replaced by the phenyl (Figure 5). The secondary structure of the mutant protein was predicted, and the helix was replaced by a sheet, which may be the reason for the dysfunction of the mutant protein (Figure 6).

So far, there are nine missense mutations of *GJA8* found in different hereditary cataract pedigrees; they are R23T, V44E, E48K, V79L, V64G, P88S, P88Q, R198Q, and I247M [5,21,23-28] (Table 2). Among these mutations, only I274M was located in the cytoplasmic COOH-terminus, where a new mutation S276F was identified in this study. I274M was found in a Russian family in 2001, and it was related to a zonular pulverulent cataract [25].

**Table 2:** Summary of identified mutations in the human connexin 50 gene.

Mutation	Amino acid change	Population	Location	Reference
68G>C	R23T	Iranian	NH_2_-terminus	5
131T>A	V44E	Indian	First transmembrane domain	23
142G>A	E48K	Pakistani	E1	24
191T>G	V64G	Chinese	E1	25
235G>C	V79L	Indian	Second transmembrane domain	27
262C>T	P88S	English	Second transmembrane domain	26
262C>A	P88Q	English	Second transmembrane domain	21
593G>A	R198Q	Indian	E2	23
741T>G	I247M	Russian	the COOH-terminus	28
827C>T	S276F	Chinese	the COOH-terminus	This paper

Autosomal dominant cataracts are morphologically and genetically heterogeneous. Similar phenotypes can map to different loci. Cataract caused by the same genetic defect in members of the same family and even in each lens of the same patient showed variable morphologies [29,30]. Further study is needed to elucidate the pathophysiological consequences of this newly identified mutation in relation to the pathogenesis of cataract. It may lead to a new therapeutic target for cataract.
